# Dual-Criteria Decision Analysis by Multiphotonic Effects in Nanostructured ZnO

**DOI:** 10.3390/mi15050579

**Published:** 2024-04-27

**Authors:** Victor Manuel Garcia-de-los-Rios, Jose Alberto Arano-Martinez, Martin Trejo-Valdez, Mónica Araceli Vidales-Hurtado, Gina Gallegos-García, Carlos Torres-Torres

**Affiliations:** 1 Sección de Estudios de Posgrado e Investigación, Escuela Superior de Ingeniería Mecánica y Eléctrica Unidad Zacatenco, Instituto Politécnico Nacional, Mexico City 07738, Mexico; 2 Escuela Superior de Ingeniería Química e Industrias Extractivas, Instituto Politécnico Nacional, Mexico City 07738, Mexico; 3 Centro de Investigación en Ciencia Aplicada y Tecnología Avanzada Unidad Querétaro, Instituto Politécnico Nacional, Santiago de Querétaro 76090, Mexico; 4 Centro de Investigación en Computación, Instituto Politécnico Nacional, Mexico City 07738, Mexico; ggallegos@cic.ipn.mx

**Keywords:** nonlinear optics, optical Kerr effect, two-photon absorption, photoconductivity, thin films

## Abstract

Simultaneous interrogation of pump and probe beams interacting in ZnO nanostructures of a two-wave mixing is proposed for dual-path data processing of optical signals by nonlinear optical effects. An enhancement in third-order nonlinear optical properties was exhibited by Al-doped ZnO thin films. Multiphoton absorption and nonlinear refraction were explored by the z-scan technique at 532 nm with nanosecond pulses. The evolution of the optical Kerr effect in the ZnO thin films was analyzed as a function of the incorporation of Al in the sample by a vectorial two-wave mixing method. Electrical and photoconductive effects were evaluated to further characterize the influence of Al in the ZnO solid samples. Potential applications of nonlinear optical parameters for encoding and encrypting information in light can be envisioned.

## 1. Introduction

The nonlinear optical (NLO) effect is an area of research that has gained great relevance in recent years due to its immediate application in various technological fields. This branch focuses on studying the phenomena that arise when high-intensity light beams obtained from laser systems interact with matter, creating multiphotonic effects (MPE). MPE occurs when the nonlinear response results in the refraction or absorption of two or more photons in response to a high-intensity light beam. The resultant energy combination exceeds its bandgap, allowing the manifestation of these effects [1]. The study of these effects allows us to visualize both real and imaginary parts of the nonlinear optical susceptibility in order to understand and determine a potential nonlinear absorption, Kerr effect, or other NLO phenomena. This consideration can be the basis for developing new technologies assisted by ultrafast phenomena. Their importance is vital in other fields, such as biomedicine, in which NLO effects have significantly contributed to applications such as high-sensitivity detection of biological functions through phase contrast [2]. In addition, MPE potential claims to exceed the limits of electronics; therefore, applications in quantum computing and high-speed optical communications using NLO have been proposed [3]. The NLO is projected as an essential component of great importance in quantum optics as researchers explore the interactions of nonlinear materials and their contribution to the creation of more robust and efficient quantum systems.

Furthermore, zinc oxide is a well-known semiconductor material due to its great potential for optoelectronic applications. The properties of ZnO make it a suitable candidate for developing highly sensitive gas sensors, dye-sensitized solar cells, field-emission devices, transparent conductive coatings, and photoluminescent materials [4]. In recent years, studies have pointed out the strong second- and third-order optical nonlinearities present in ZnO, which have found applications as integrated NLO devices, such as nonlinear propagation in fibers, optical modulators, optical limiting, and optical switches [5].

The basic characteristic properties of pure ZnO thin films can be controlled and improved by doping them with chemical elements from group III transition metals, which endows them with better free carrier density, electric conductivity, and a high optical UV transition spectrum [6]. From these elements, the selection of aluminum as dopant has demonstrated itself to be the best candidate due to its low cost [7], easy incorporation [8], and compatibility [9]. Al can be an excellent dopant for improving the physical properties exhibited by ZnO. By adding a single positive Al^3+^ to a ZnO sample, the resulting ionic radius in the system can produce stresses and defects that have a strong influence on the structural, electrical, optical, linear, and nonlinear properties of the crystal [10]. The inclusion of Al in ZnO substrates can be considered an attractive alternative and suitable to occupy particular interstitial sites, resulting in changes in transparency, optical transmittance, and nonlinear optical properties [11]. The Al dopant incorporation creates oxygen vacancy defects in the ZnO structure, which shifts the energy band gap (*E_g_*) from 3.12 to 4.30 eV [6], directly reflecting an enhancement in the inner properties. Al-doped ZnO films, known as AZO, have attracted the attention of the research community for their interesting, enhanced properties and large surface/volume ratio, which make them favorable for the fabrication of various nanometric optoelectronic and optical devices. They also serve as a cheaper alternative to indium tin oxide (ITO) transparent electrodes [12]. Diverse scientific reports have pointed out their exceptional high electrical conductivity [13,14], which has been proven to affect the NLO behavior. The incorporation of dopants in the inner structure of ZNO may be directly linked to its little-explored properties, such as second- and third-harmonic generation [15]. The analysis of the electrical and optical properties exhibited by AZO films promises to be useful in the production of nanostructured materials in solar cells [16], along with other optoelectronics and optical devices like acoustic wave surfaces, photoelectrodes, flexible displays, optical waveguides, window layers, display technology, and transparent electrodes for photovoltaic functions [17].

The possibility of exploiting the great potential of non-expensive AZO materials to generate a variety of all-optical systems sounds very attractive. With this motivation, this research is mainly devoted to analyzing nanosecond, third-order nonlinear optical effects that arise from the incorporation of Al doping in ZnO thin solid film. Experimentation by means of a z-scan technique was employed to visualize the energy transmitted through the sample in order to guarantee the enhancement of the nonlinearity by the inclusion of Al dopants in the ZnO. This easy-to-assemble experiment allowed us to obtain precise information about the nonlinear behavior taking place due to the high irradiation. A TWM was employed to determine the nonlinearity for the dual-decision analysis design. The high irradiance of the beams induced a transformation of the polarization state that fomented a change in the polarization of both beams, which consequently affected their transmittance [18]. Also, standard characterization of the structure, morphology, electrical behavior, and photoconductive effects was undertaken. The polarization-selectable nonlinearities promoted by vectorial two-wave mixing allowed us to highlight the potential of nonlinear optical phenomena for controlling and processing signals in dual-path logic decision-making. The basis of our main findings seems to be a good candidate for developing all-optical functions governed by nanoscale nonlinear materials.

## 2. Materials and Methods

### 2.1. Synthesis and Characterization of ZnO and AZO Thin Films

The ZnO nanostructures were synthesized in thin film form using a spray pyrolysis technique. We employed a precursor solution composed of 2.65 g of zinc acetylacetone (Zn(C_5_H_7_O_2_)_2_) (0.01 mol) mixed with 12.54 mL of deionized water, 83.88 mL of methanol (CH_3_OH), and 3.58 mL of acetic acid (C_2_H_4_O_2_). On the other hand, for the fabrication of AZO thin films, we used another precursor solution that consisted of 2.6396 g of zinc acetylacetone (0.01 mol) and a doping of 0.0996 g of aluminum acetylacetone (Al(C_5_H_7_O_2_)_3_), making sure to add 0.0003 mol to the mixture. Next, 12.54 mL of deionized water, 83.88 mL of methanol, and 3.58 mL of acetic acid were employed following a similar reference method to obtain the correct proportions [19]. Both solutions were stirred for about 10 min, and around 15 layers of material were deposited on preheated 10 × 25 × 1 mm SiO_2_ substrates. These substrates were ultrasonically cleaned with ethanol for 20 min and then with deionized water to be preheated at 430 °C on a graphite surface over a fused tin bath. The deposition was achieved by nebulizing the precursor solution on the substrates with a vapor exposition of 180 s at a fixed input pressure of 7 L/min and output of 3 L/min, with a cooling time of 10 min between deposits.

The sample morphology was characterized by a dual-beam microscope (Nova200 Nanolab, Beijing, China) and scanning electron microscope (SEM; SEM ULTRA 55 FEG System from ZEISS with Secondary Electron and Backscattering Detector, Graz, Austria) in order to obtain representative images for the analysis of a considerable portion of the surface and obtain their morphology. This also made possible the visualization of the sample porosity, thickness, and crystal orientation on the surface. Moreover, optical absorbance spectra were obtained using a UV spectrophotometer (Perkin Elmer XLS, Waltham, MA, USA) for both the ZnO and AZO samples. Furthermore, in order to determine the energy band gap of the studied semiconductor samples, we calculated the Tauc plots using the previously obtained absorbance spectra. The Tauc method is based on the assumption that the energy-dependent linear absorption coefficient α can be expressed by the following equation [20]:

(1) $${\left(\alpha h\nu \right)}^{\gamma }=A(h\nu -E_{g}),$$

where h is the Planck constant, ν is the photon’s frequency, $E_{g}$ is the band gap energy, and A is a proportionality constant. Also, the γ factor denotes the nature of the electron transition and is equal to 1/2 or 2 for the direct and indirect transition band gaps, respectively.

### 2.2. Electrical and Photoconductivity Studies

In order to analyze the photoconductivity response in the two thin films, experiments using an Autolab potentiostat (Autolab/PGSTAT302N high-power potentiostat/galvanostat, METROM, Herisau, Switzerland) were carried out to observe the effects of variable frequencies, from 1 to 100 kHz, on the sample impendance. A pair of copper electrodes was placed on both edges of the studied samples in order to obtain reliable data on the measured impendance. Also, using light irradiation from a UVP PenRay^®^ lamp model 90-0012-01 (11SC-1), it was possible to induce photoconduction as an impendance change that exists during the irradiation time. The data were collected using the Metrohom Nova 1.1 software, which also allowed us to interpret and graph the obtained results. The mathematical expression used to analyze the conductivity increase (∆σ) induced by the UV exitation in semiconductors is described by the following equation [21]:

(2) $$\Delta \sigma =e(\Delta n\mu_{n}+\Delta p\mu_{p}),$$

where e is the electron elementary charge 1.602 × 10^−19^ (coulombs), and Δn is the relative change of the number of electron-hole pairs with the induced light. Due to the charge neutrality assumed to be maintained during illumination, Δp=Δn, which describes the relationship between the number of electrons ($n_{e}$), holes ($n_{h}$), and the fundamental electric charge in an intrinsic semiconductor. Finally, $\mu_{e}$ and $\mu_{h}$ are the electrons and hole mobilities, respectively.

### 2.3. Third-Order Z-Scan NLO Characterization

**Figure 1:**
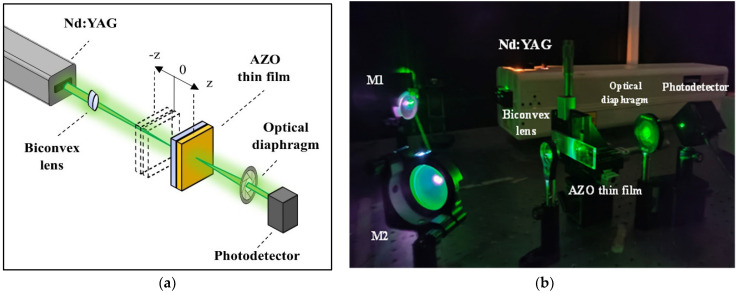
The z-scan experimental setup: (**a**) scheme, (**b**) photograph.

Irradiation from a Nd:YAG high-powered laser (Continuum SL II-10, Cambridge, MA, USA) at 532 nm and a pulsed time of 4 ns were used to characterize the NLO properties of both samples. The study of multiphoton absorption and refraction was made possible by assembling a z-scan configuration in a range between 4 and −4 mm to quantify the transmittance and then calculating a possible Kerr or multiphoton absorption effect. A CS_2_ sample was employed to calibrate the z-scan system. The experimental setup is illustrated in Figure 1.

In order to estimate the nonlinear optical parameters, an approximation of the optical transmittance $T_{0}$ in ZnO using the open configuration can be determined using the following equations [22]:

(3) $$T_{0}(z,\Delta \Phi_{0})=1-\frac{\left(\beta I_{0}L_{eff}\right)}{\left(2\sqrt{2}(1+Z^{2}/Z_{0}^{2})\right)},$$

(4) $$L_{eff}=\frac{\left(1-e_{0}^{\left(-\alpha L\right)}\right)}{\alpha },$$

where β denotes the nonlinear absorption coefficient, $L_{eff}$ represents the effective length, and L represents the sample length. In addition, for analyzing the closed configuration using a Gaussian beam of radius $w_{0}$, the normalized transmittance $T_{c}$ as a function of position z was calculated as follows [23]:

(5) $$T_{c}(z,\Delta \Phi_{0})=1-\frac{\left(4\Delta \Phi_{0}(Z/Z_{0})\right)}{\left(Z^{2}/(Z_{0}^{2})+9)(Z^{2}/(Z_{0}^{2})+1\right)},$$

(6) $$\Delta \Phi_{0}=k\Delta n_{0}L_{eff},$$

where $Z_{0}=kw_{0}^{2}/2$ and k=2π/λ, with λ being the optical wavelength, $∆\Phi_{0}$ the optical phase change, and $\Delta n_{0}$ the refractive index change of the product of the nonlinear refractive index ($n_{2}$) and the peak focus irradiance on the propagation axis ($I_{0}$).

### 2.4. Dual-Criteria Decision Analysis by a TWM Method

A TWM method can be employed in order to explore the vectorial nature of the nonlinear optical behavior of transmitted beams. The simultaneous analysis of the multiphotonic absorption in the pump beam and the nonlinear refraction in a probe beam in a TWM interaction where both beams intersect each other, creating interference patterns, was proposed for designing a dual-criteria decision function. This method is based on the interrogation of the irradiance induced by different polarization angles on the initial incident beams because they are constants in the TWM system.

The TWM can be described as an intermodulation of two coherent waves that are overlapped in an optical medium [24]. This experiment was carried out with two beams with the same wavelength in order to explore the vectorial nature of the induced birefringence, which emerges from the superposition of the irradiance and polarization of the beams in the interaction. When both incident beams intersect at a material surface featuring high irradiance, they propagate with the influence of a nonlinear refractive index and with the contribution of a nonlinear optical absorption. In our experimental setup, there was a half-wave plate that dynamically rotated the polarization of the probe beam. The gradual rotation induced by the half-wave plate on the probe propagation was conducted every 5 degrees, from 0 to 90, generating an optical nonlinearity depending on the contrast of the fringe pattern resulting from the interference of the incident beams. During the rotation, the maximum Kerr transmittance can be expected at an angle of polarization of 45° between the beams. The inducted birefringence can originate from different physical mechanisms of optical nonlinearity, like thermal processes, electronic polarization, electrostriction, and molecular orientation.

The proposed method for the generation of the dual-criteria analysis is based on the interrogation of the irradiance induced by different angles of polarization due to rotation on the incident beams. In our TWM setup, the polarization of the pump beam was fixed, while the polarization of the probe was able to rotate, allowing it to have polarized components that were either orthogonal or parallel with respect to the plane of polarization of the probe beam. The transmitted irradiance detected in this interaction was normalized and considered as numerical inputs for a dual-criteria decision-making design.

The high-intensity irradiation from the Nd:YAG optical source on the studied materials generated MPE, which was confirmed by the z-scan measurements. The inclusion of Al dopants in ZnO had the objective of enhancing the nonlinear optical response, for the refractive index and for the multiphotonic absorption. On the other hand, the TWM allowed the observed transmittance to be controlled depending on the third-order nonlinear optical effects [25].

**Figure 2:**
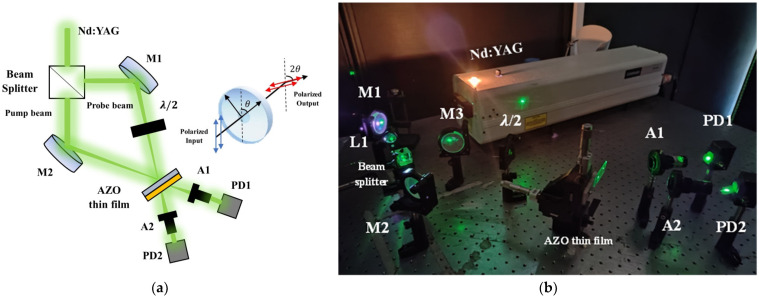
TWM experimental setup: (**a**) scheme, (**b**) photograph.

As illustrated in Figure 2, the experimental setup implemented by our Nd:YAG laser system emitted a 532 nm wavelength focused on 1 mm^2^ on the analyzed sample. A 50 mm optical biconvex lens was employed for focusing the linearly polarized beam. The pump and probe beams of the interaction emerged from a beam splitter and intersected at the sample interface. Assuming both beams were symmetrical, the angle θ between them was estimated to be 60°. L1 is an optical lens, M1 and M2 are mirrors, and λ/2 denotes the half-wave plate. Two polarizers (A1 and A2) were employed to analyze the orthogonal polarized transmitted probe beam and the parallel polarized transmitted pump beam with respect to the incident polarizations. The irradiance data were recorded by a pair of PIN photodetectors (PD1 and PD2) and an ADS1102CAL ATTEN digital oscilloscope.

The transmitted irradiance of the TWM interaction can be described by the wave equation [26]:

(7) $$\nabla^{2}E_{\pm }=-\frac{n_{\pm }^{2}\omega^{2}}{c^{2}}E_{\pm },$$

where $E_{\pm }$ represents the electric fields that propagate in their components $E_{+}$ and $E_{-}$, and ω is the optical frequency. The refraction index is n, and c denotes the speed of light in a vacuum. The nanostructured surfaces on the analyzed samples were composed of disordered crystal formations. However, due to the analyzed sample dimensions, both materials were considered isotropic. This assumption was later verified by experimentation with the nonlinear optical response. In this experiment, we considered the minimum possible angle between the propagating vectors of the beams in order to analyze potential changes in Kerr transmittance as a function of the rotation of the sample with the beams near normal incidence. Also, we considered a circular polarization of the pump beam, which allowed the orthogonal component of the probe beam to be phase matched [27]. In addition, it is worth mentioning that inducing a change in the angle between the beams can produce a totally different phase matching in the system, which strongly affects the third-order nonlinear optical phenomena originating from these interactions. As for the TWM system, only the propagation plane of the irradiated beams was polarized, which greatly enhanced the optical nonlinearity of the materials without affecting the phase-matching condition of the experiment. The refractive index in a nonlinear medium can be considered irradiance dependent and described by the following equation:

(8) $$n_{\pm }^{2}=n_{0}^{2}+4\pi (\chi_{1122}^{\left(3\right)}|E_{\pm }{|}^{2}+(\chi_{1122}^{\left(3\right)}+\chi_{1212}^{\left(3\right)})|E_{\pm }{|}^{2}),$$

where $n_{0}$ is the refractive index, and $\chi_{1122}^{\left(3\right)}$ and $\chi_{1212}^{\left(3\right)}$ are the independent components of the third-order optical susceptibility tensor of the system. Furthermore, the mathematical expression used to obtain the transmitted irradiance I in terms of the propagation distance L and the incident irradiance $I_{0}$ is as follows:

(9) $$I(L)=\frac{I_{0}\exp\left(-\alpha_{0}L\right)}{1+\beta I_{0}L_{eff}},$$

## 3. Results

### 3.1. Morphological Characterization and UV–Vis Evaluation

**Figure 3:**
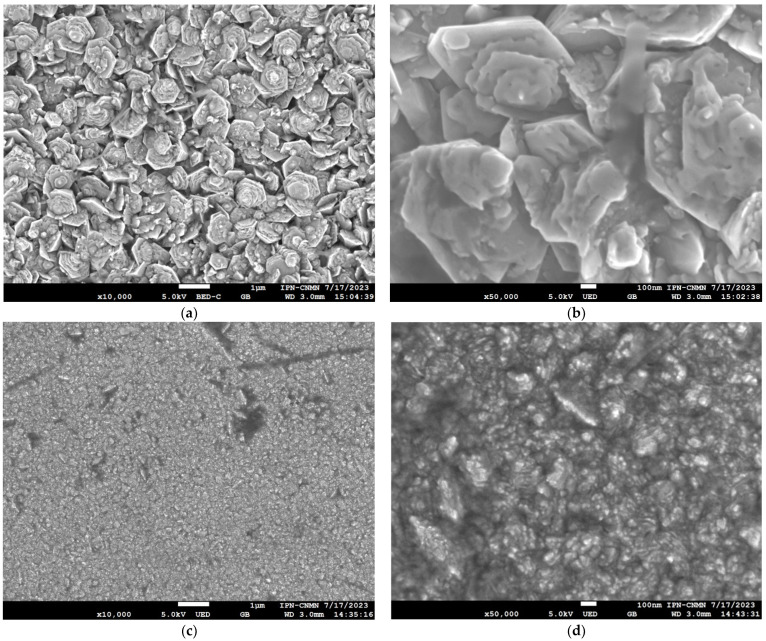
SEM images of the studied samples. The surface morphology of the ZnO samples at magnification scales of (**a**) 1:1 μm and (**b**) 1:100 nm. The surface morphology of the AZO samples at magnification scales of (**c**) 1:1 μm and (**d**) 1:100 nm.

The results depicted in Figure 3 show the SEM images of the surface of the films that demonstrate the formation of homogenous nanoflake-like crystals that conform to the ZnO nanomaterial. The measured flake thickness was estimated to be approximately in the range of 30 to 150 nm. In addition, the AZO film showed a nanometric agglomeration of the particles, whose spherical and elliptical clusters were estimated to be between 10 and 300 nm long. This could be explained by the nucleation of Al atoms with the differences in ionic Al (0.39 Å) radius with Zn (0.60 Å), which may be responsible for the reduction in the grain size when crystallization takes place.

Studies by ellipsometry were carried out to obtain relevant data for characterizing the nanoscale thickness of both films. The data revealed a material deposition of approximately 688 and 776 nm for the ZnO and AZO films, respectively, attributed mainly to the synthesis technique and number of deposits utilized to obtain both materials. These microscopy images showed the significant doping effect of ZnO.

**Figure 4:**
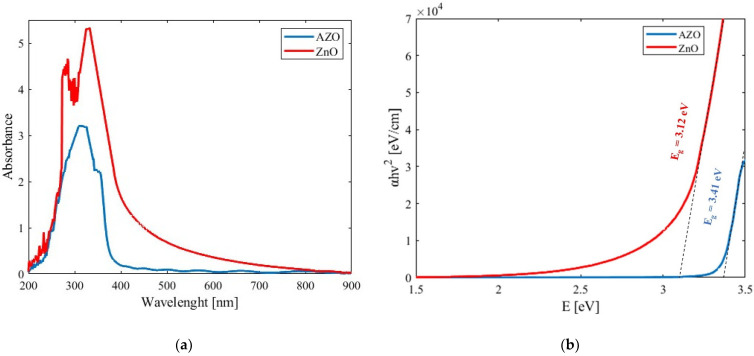
(**a**) UV–Vis data obtained for the ZnO and AZO samples. (**b**) Tauc plots for obtaining the energy bandgap of the studied samples.

The absorbance exhibited by the ZnO and AZO thin films obtained by UV–Vis spectrometer is shown in Figure 4a. From the experimental data, it can be seen that there was a preference for low-frequency UV absorbance at wavelength regions ranging from 200 to approximately 400 nm. In the case of the AZO samples, the absorbance decreased. Also, from the obtained data, it was possible to calculate the Tauc plot, as shown in Figure 4b. The bandgap energy of the samples was determined to be about 3.12 and 3.41 eV for the ZnO and AZO films, respectively. In a similar way, by taking the absorbance values, it was possible to calculate the absorption coefficients for each studied sample. The estimated values of α were close to $1.85\times 10^{5}$ and $9.054\times 10^{4}\;{\mathrm{cm}}^{-1}$ for ZNO and AZO, respectively.

The decrease in the absorbance of the doped film can be explained by the ionic substitution of Al_3+_ atoms at the Zn_2+_ sites, which degenerates optical absorption. A decrease in optical absorption with doping increments has been reported in the literature [28]. Moreover, the decrease in absorbance with increasing Al dopant concentration indicates a widening of the band gap. Also, the change in optical absorption at higher doping levels may be attributed to a shrinkage effect due to the decrease in size of the nanoparticles, which in turn increases their porosity and ultimately widens the band gap [29].

It is worth mentioning that a similar shift of *E_g_* in pure ZnO and Al-doped materials has been reported. This behavior can be explained by the Moss–Burstein effect [30]. This effect implies that each Al ion is bound with oxygen ions, and the left one exceeds electrons, which pushes the Fermi energy level to a higher energy level, leading to an increase in charge carrier concentration and the creation of an n-type semiconductor. It is possible that the nanoparticle contribution of Al in the ZnO may be responsible for a higher bandgap shift than the nanoflake-like structures; it can also be explained by the same shrinkage effect due to the particle size.

### 3.2. Electrical and Photoconductive Studies

**Figure 5:**
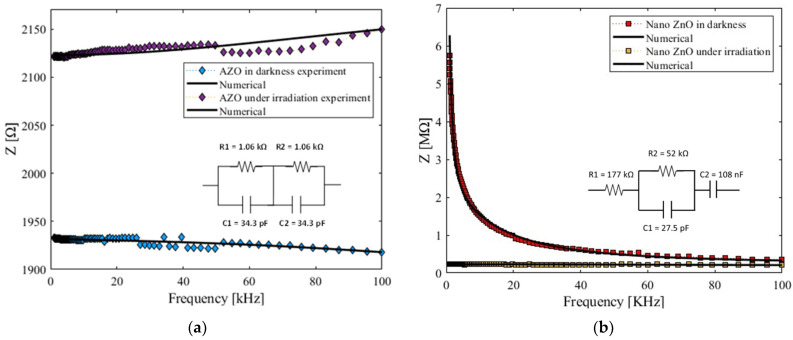
Electrical impedance as a function of frequency for the (**a**) AZO samples and (**b**) ZnO samples studied and their respective best-fitting electrical RC circuits.

Figure 5 presents the electrical impedance (Z) as a function of the electrical frequency in the ZnO and AZO samples. These results are indicative of the conductive nature of the semiconductor and resemble a persistent photoconduction (PPC) build-up effect; remarkably, the mentioned behavior seems to be purely thermal. A comparison of the effects exhibited by the sample showed a capacitive behavior of the electrical response of the sample in darkness for most of the spectrum explored. The change in the resistive behavior of semiconductors could be attributed to the density of the charge carriers that were excited by the irradiation of light, as can be seen in Figure 5.

In addition, using the Nova 1.1 software, it was possible to obtain the equivalent electrical RC–RC parallel circuit, which represents the best numerical convergent fit with minimal error (0.0018). Typical models representing high-capacity ion batteries [31] have this established configuration. Also, the proposed circuit, known as a bricklayer model (BLM), is commonly used for the analysis and electrochemical impedance characterization of polycrystalline ceramics [32].

### 3.3. Nonlinear Z-Scan Measurements Results

**Figure 6:**
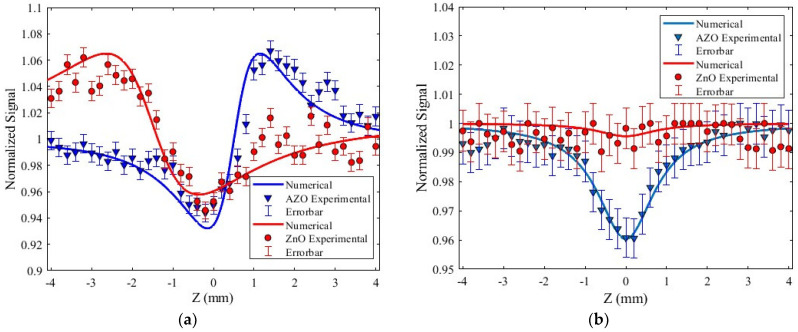
The z-scan results for the ZnO and AZO sample: (**a**) closed aperture, (**b**) open aperture.

The z-scan measurements were undertaken for the determination of the nonlinear absorption and nonlinear refraction, as presented in Figure 6a,b. From the results, it can be deduced that self-focusing is associated with a positive Kerr effect induced by an irradiance of 6.77 GW/cm^2^ in AZO. Also, using the open aperture configuration, it was found that there was a two-photon absorption at the same irradiance. By comparing the ZnO nanostructured sample, a self-defocusing effect and a very small nonlinear absorption index were measured. The approximate nonlinear parameters for the AZO sample corresponded to $n_{2}=7.4\times 10^{-12}\;{\mathrm{cm}}^{2}/W$ and $\beta =1.7\times 10^{-7}\;\mathrm{cm}/W$, while those for the ZnO sample corresponded to $n_{2}=-1.36\times 10^{-11}\;{\mathrm{cm}}^{2}/W$ and a negligible nonlinear absorption within the error bar.

The contrast in the $n_{2}$ sign can be explained considering a different physical energy transfer mechanism generated by multiphoton processes under thermal and electronic optical effects. The results in Figure 6a indicate that the introduction of Al dopants to the ZnO structure gave rise to an enhancement in the nonlinear Kerr response. Also, from Figure 6b, it can be observed that there was an enhancement in the multiphotonic absorption using AZO thin films.

Comparable works have reported NLO parameters in ZnO thin films by RF magnetron sputtering, whose thicknesses were 500 and 1200 nm with reported values of $\beta =4.74\times 10^{-4}\;\mathrm{cm}/W$ and $\beta =2.59\times 10^{-4}\;\mathrm{cm}/W$, respectively [33]. Also, a ZnO thin film sample with a thickness of 425 nm synthesized by atmospheric pressure chemical vapor deposition (APCVD) was used in z-scan measurements to observe the influence of different irradiances ranging from 166 to 218 W/cm^2^ [34]. The results indicated a notable difference with variable irradiance, with β values ranging from 9.2039 to 10.5511 cm/W and $n_{2}$ values ranging from $0.101\times 10^{-3}$ to $0.18\times 10^{-3}\;{\mathrm{cm}}^{2}/W$. In a similar way, the refractive index and nonlinear absorption coefficient of ZnO nanoparticles have been measured by similar z-scan measurements, obtaining values of $n_{2}=6.59\times 10^{-9}\;{\mathrm{cm}}^{2}/W$ and $\beta =1.75\times 10^{-3}\;\mathrm{cm}/W$, respectively [35].

Furthermore, in z-scan experiments on Al-doped ZnO thin films, for instance, a 3% wt doped sample grown by spray pyrolysis was irradiated at different radiation doses (5–20 kGy) [36]. The results showed no correlation between the irradiation and its nonlinear properties, obtaining β values varying from $4.65\times 10^{-2}$ to $10.9\times 10^{-2}\;\mathrm{cm}/W$; however, a sign change in the nonlinear refractive index was reported using this material, with values ranging from $-5.60\times 10^{-2}$ to $-26.8\times 10^{-2}\;{\mathrm{cm}}^{2}/W$. Similar studies using z-scan experiments at 1064 nm on AZO thin films have been conducted for developing nonlinear photonics applications in the ns regime [37], and the obtained results exhibited a large negative nonlinear refractive index of $n_{2}=-5.48\times 10^{-9}\;m^{-13}/W$. Also, comparable AZO thin films at 3% wt have been measured by a z-scan technique, with the results showing $\beta =2.34\times 10^{-4}\;\mathrm{cm}/W$ and $n_{2}=8.65\times 10^{-8}\;{\mathrm{cm}}^{2}/W$; that article also indicated a sign change from negative to positive [38]. Furthermore, it has been reported that Al-doped ZnO can present $n_{2}$ values ranging from −2.30 to $0.645\;{\mathrm{cm}}^{2}/W$; similarly, β values ranging from $4.51\times 10^{-6}$ to $1.72\times 10^{-6}\;\mathrm{cm}/W$ were obtained, with the variation attributed to the plane orientation [39].

### 3.4. Dual-Criteria Decision Analysis by a TWM Method

**Figure 7:**
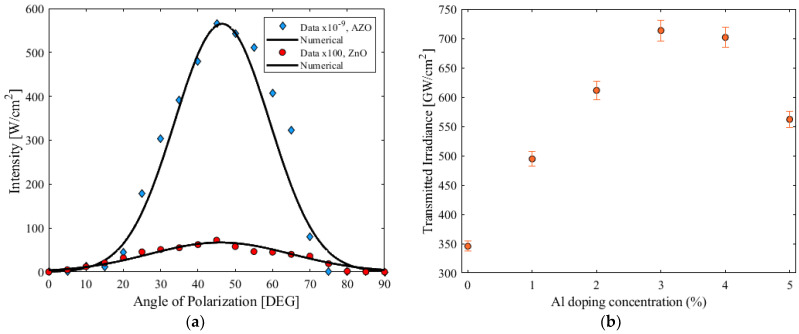
(**a**) Transmitted probe irradiance as a function of the angle of polarization in the TWM studied. (**b**) Transmitted probe irradiance as a function of the Al doping concentration of the AZO thin films.

In Figure 7a, the transmitted probe beam is plotted as a function of the angle of polarization obtained by the TWM experiment. The change in refractive index can be attributed to the accumulated photoenergy that produces heat. Therefore, the numerical simulation using Equation (8) describes the change in nonlinear refraction due to a particular temperature change. For this investigation, the fitting curve shown in Figure 7a corresponds to 0 for parallel and orthogonal polarizations in the interaction, which implies that $\chi_{1122}^{\left(3\right)}=0$. With these results, we assumed a thermal process as the main physical mechanism responsible for the present nonlinearity [1].

In Figure 7b, the transmitted probe irradiance is plotted as a function of the Al doping concentration of the AZO thin films. It can be observed that there is a clear enhancement of the Kerr effect due to the incorporation of Al in the ZnO.

The results showed that the NLO response did not increase monotonically by adding higher concentrations of Al dopant, and this was the reason an experiment was carried out in order to see the reaction between the volume fraction of dopant and the nonlinear irradiance, from 0% (pure ZnO) up to 5%. The information revealed a maximum transmittance at 3% of dopant content for this investigation. These results are in good agreement with similar works that presented a degradation effect in Al-doped ZnO thin films with much higher incorporation of the dopant [40,41].

In order to demonstrate the potential dual-criteria decision analysis that can be performed by a TWM method, we simultaneously measured the transmittance of the pump and probe beams, considering that the transmission axes of analyzers A1 and A2 were parallel and orthogonal to the plane of the polarization of the incident beams. The results are shown in Table 1.

The contrast in the behavior of the pump and probe beams depending on the input signal confirms an active redundancy system. The z-scan measurements in Figure 6a demonstrate the nonlinear phenomenon. The fluctuation in the nonlinear refraction induced by the second harmonic of the laser system confirms the nonlinear enhancement due to the Al doping. In addition, the induced transmitted irradiance shown in Figure 7a proves the birefringence induced by the TWM, which can also be modulated as a function of the Kerr effect and the nonlinear optical absorption. Consequently, an irradiance-dependent refractive index in an anisotropic material represents the potential control of the Kerr transmittance and birefringence in the nonlinear optical medium [42]. This is possible using the proposed TWM experiment and rotating the propagation of the weakly induced beam. Simultaneously, the pump beam is depleted by the two-photon absorption that emerged from the increase in intensity, with an irradiance fringe pattern derived from the superposition of the beams in the ZnO. On the other hand, when the linear polarization of the beams is mutually orthogonal, no transmission of the probe beam is present, and no important depletion of the pump beam occurs.

Certainly, implementing an MPE on a multi-decision function platform sounds very interesting for diverse applications. The question remains as to whether other dual- or multi-decision-making designs could be applied to other materials that have reported different MPEs by varying the pulsed radiation, doping material, concentrations, and even optical wavelengths. Recently, it has been reported that free carrier absorption, excited-state absorption, and nonlinear scattering influence the NLO properties exhibited in ZnO materials [43]. This paves the way for potential applications for optical power limiting [44] and other photovoltaic functions [45] that could be controlled by MPE.

Moreover, the influence of different ns pulsed irradiations in Cu/Ag/AZO multilayer films has been investigated to improve the optical and electrical properties of films [46]. In contrast, saturable absorption has been measured in ZnO films at 1560 nm [47], which can be tailored during the processing route of the synthesis [48]. Moreover, it has been pointed out that an increase in light scattering is necessary to obtain a higher transmittance in AZO films [49]. Irradiation on AZO thin films by fs and ns laser pulses can induce a change in nonlinear transmittance [50] that can be controlled as a function of irradiation exposure time [51]. Some of these changes in transmittance have been attributed to interband carrier dynamics in AZO thin films [52,53,54]. This work points out the importance of NLO phenomena for engineering all-optical functions, with potential applications for developing low-dimensional functions, all-optical nanoscale devices, and multi-decision decision analysis platforms.

## 4. Conclusions

An all-optical redundancy system based on a TWM experiment was demonstrated, with potential applications for ultrafast dual-criteria decision functions. A strong enhancement in the nonlinear optical response exhibited by nanostructured ZnO was obtained by the incorporation of particular concentrations of Al dopants. A shift in the bandgap and an increase in the transparency of the ZnO nanostructures were promoted by the electronic effects resulting from the inclusion of Al in the ZnO sample in thin film form. Electrical and photoconductive studies showed capacitive behavior that can be modulated by optical irradiation in the samples. The contrast in the sign of the nonlinear refraction of ZnO and AZO at a 532 nm wavelength, together with the inhibition of the two-photon absorption as a function of the presence of Al in ZnO, seems attractive for designing nonlinear optical platforms for signal processing.

## Figures and Tables

**Table 1:** Experimental data for dual-criteria decision analysis by a TWM method.

Input Digital Signal	Angle of Polarization between the Pump and Probe Beam [DEG]	Normalized Transmitted Pump Beam	Normalized Transmitted Probe Beam	Transmitted Pump Beam [GW/cm^2^]	Transmitted Probe Beam [W/cm^2^]
0	45	0	1	2.89	550
1	90	1	0	3.01	5

## Data Availability

Data and materials are available upon reasonable request to C. Torres-Torres (ctorrest@ipn.mx).
